# Progress in Surgery

**Published:** 1899-03-11

**Authors:** 


### PROGRESS IN SURGERY.

### OTOLOGY.

**Otitis Media.**—The importance of sterilising instruments used in the practice of otology needs emphasising, and the hints given by McKee[1] on asepsis in otology and laryngology are worthy of note: (1) To sterilise cutting instruments place in 2½ per cent. carbolic solution for 15-20 minutes; then dip for a few seconds in boiling water. (2) To sterilise trays or dishes for instruments pour over them a few drops of alcohol and ignite. The asepsis is complete. (3) To sterilise blunt instruments (forceps, spatulæ, &c.), pass through a spirit lamp which should be on every instrument table. (4) To sterilise catheters, antrum cannulæ, &c., boil for a few moments in a porcelain-lined dish. (5) To sterilise cotton pledgets for wiping out the ear, dip the cotton wound probe into a saturated alcoholic solution of boracic acid, ignite it and allow it to burn for a few seconds, extinguishing before the cotton is charred. (6) Needles preserve in pure lysol.

Ewing[2] also protests against the practice of douching suppurating ears since moisture favours the growth of pathogenic micro-organisms, and he cites in illustration the fact that a traumatically ruptured drum membrane will seldom suppurate if kept dry. He recommends iodoform gauze or bichloride or borated gauze for packing the ear instead. Milligan[3] in a recent paper reminds us that Lermoyez regards the invasion of staphylococci from without as the invariable cause of chronic suppurative middle ear disease. Milligan states that whether this be so or not there is much to be said for Pes and Gradinego's aseptic method of treatment, which consists in sterilisation of the auricle and meatus, the introduction of iodoform gauze into the auditory canal, and the application of a sterilised pad over the external ear; in other words, an attempt to prevent the entrance into the middle ear of germ life from without. This method has the additional advantage of keeping the parts fairly dry, and the abstraction of fluid is undoubtedly prejudicial to the activity of germ life.

In the course of a more recent paper on intracranial suppuration of otitic and rhinitic origin, Milligan[4] discussed the various paths along which infection might be conveyed from the middle ear to parts within the cranium. He especially dwelt on the dangers of bony erosions in and around the middle ear and the neighbouring mastoid cells. The routes along which pathogenic organisms might travel are: (1) Minute venous trunks; (2) minute lymphatic trunks; (3) periarterial fibrous sheaths; (4) perineural fibrous sheaths; (5) fibrous septa running between the tympanic mucosa and the dura mater; (6) thrombosed vessels running through the imperfectly ossified petrosquamosal and petro-mastoid suture.

**Middle Ear Suppuration.**—De Santi[5] draws attention to the fact that of all the diseases of the ear that come under the notice of the aural surgeon, by far the most frequent is purulent inflammation of the middle ear. The presence of a discharge of the ear or ears is often looked upon with complete indifference by patients or their parents, and even not infrequently by their medical advisers, and the consequences and complications are often grave. He deals with the subject under two headings—1, the stage before perforation; 2, the stage after perforation.

1. The stage before perforation: The symptoms present in this stage may be very severe—acute otitis media purulenta—or, on the other hand, slight and ill-marked, subacute and leading to painless perforation. When severe they are as follows: Excruciating pain in and around the ear and head, causing the patient to scream out, and sometimes, especially in children, ending in delirium, vertigo, raised temperature (101 to 103 deg. Fahr.), quick pulse, and furred tongue. Convulsions and coma occasionally occur. Deafness is more or less marked, but in the presence of the severe pain is probably not noticed. The external meatus may be swollen, red, and very tender. In young children the intense pain in the head, the screaming, delirium, and fever may lead to a diagnosis of meningitis; the onset of the latter is, however, usually not so sudden, the pulse and respirations are different, and cerebral vomiting is absent; there are also usually no eye symptoms, such as optic neuritis. The appearances of the membrane, as seen through the speculum in the early stage before perforation, are those of hyperæmia of varying intensity, the injection of the cutaneous vessels being most marked at the periphery of the membrane, the pars flaccida, and the handle of the malleus; in some cases of more acute nature the whole of the drum becomes of a bright red colour. Later, as the inflammation increases, exudation occurs and pus is secreted. There is now seen to be a general or localised bulging of the swollen membrane, such saccular bulgings being usually of a yellowish green or red colour. Purulent exudation may, however, be present in the tympanic cavity without any bulging of the membrane, the latter having been altered and thickened previously. The superficial structures over the mastoid may be swollen, and the glands below and behind the ear enlarged and tender. The duration of the first stage is variable; rupture as a rule takes place in from three to five days, and is commonly followed by cessation of the pain, and more or less profuse discharge.

